# Duchenne muscular dystrophy–like phenotype in an LGMD2I patient with novel *FKRP* gene variants

**DOI:** 10.1038/s41439-020-0099-x

**Published:** 2020-04-20

**Authors:** Tetsuya Okazaki, Kaori Matsuura, Noriko Kasagi, Kaori Adachi, Masachika Kai, Mariko Okubo, Ichizo Nishino, Eiji Nanba, Yoshihiro Maegaki

**Affiliations:** 1 Division of Clinical Genetics, Tottori University Hospital, Yonago, Japan; 2 Research Initiative Center, Organization for Research Initiative and Promotion, Tottori University, Yonago, Japan; 3 Technical Department, Tottori University, Yonago, Japan; 4 Department of Neuromuscular Research, National Institute of Neuroscience, National Center of Neurology and Psychiatry, Tokyo, Japan; 5 Research Strategy Division, Organization for Research Initiative and Promotion, Tottori University, Yonago, Japan; 6 Faculty of Medicine, Division of Child Neurology, Department of Brain and Neurosciences, Tottori University, Yonago, Japan

**Keywords:** Genetic testing, Neuromuscular disease

## Abstract

A 32-year-old man initially received a diagnosis of Duchenne muscular dystrophy (DMD). Genetic analysis revealed two novel heterozygous *FKRP* variants: c.169G>A (p.Glu57Lys) and c.692G>A (p.Trp231*). These results indicated that the patient had limb-girdle muscular dystrophy type 2I (LGMD2I) caused by recessive *FKRP* variants. Patients with LGMD2I and DMD have many overlapping phenotypes. LGMD2I should be considered in patients who have a DMD phenotype but not a DMD pathogenic variant.

## Introduction

The gene *FKRP* on chromosome 19q13.3 encodes a fukutin-related protein, which is necessary for posttranslational glycosylation of alpha-dystroglycan. In particular, the normal sugar chain contains tandem structures of ribitol-phosphate, and fukutin-related protein encodes an essential enzyme for the synthesis of this structure^1^. *FKRP*-related disorders appear to result from impairment of the *O*-mannosyl glycosylation pathway of alpha-dystroglycan^2^. Patients with *FKRP* variants have several phenotypes, including limb-girdle muscular dystrophy type 2I (LGMD2I), muscle–eye–brain disease and Walker-Warburg syndrome. Approximately 100 *FKRP* variants have been identified, of which 60 cause LGMD2I^3^. LGMD2I is an autosomal recessive disorder and is clinically characterized by progressive muscle weakness with proximal-dominant muscle involvement, hyperCKemia and cardiac and respiratory failure. The c.826C>A variant (p.Leu276Ile) is most frequent among patients with LGMD2I, whose phenotype is similar to that of Duchenne muscular dystrophy (DMD). In addition, patients with LGMD2I caused by other variants have been described in previous reports as having DMD-like phenotypes^4^. The prevalence of dystrophinopathy (DMD and Becker-type muscular dystrophy) has been reported^5^ to be ~2 per 10,000, whereas the prevalence of LGMD2I is 4.3 per million^6^. Thus, many physicians might not be familiar with LGMD2I. We report the case of a 32-year-old man with LGMD2I who was initially diagnosed with DMD, which could provide useful information for an early diagnosis in patients.

The man had been born to nonconsanguineous parents at 40 weeks of gestation. No family history of neuromuscular diseases or motor developmental delay was reported. There was no birth asphyxia. He began to walk at 12 months of age. When he was 1 year and 1 month old, asymptomatic elevations of serum creatine kinase (6700 mg/dL) were noticed. Muscular specimens at the age of 1 year and 3 months showed dystrophic muscular phenomena. Dystrophin immunostaining was not available at that time, and the clinical diagnosis was Duchenne muscular dystrophy. At 7 years of age, he began having trouble climbing stairs. At 10 years of age, he had difficulty walking and needed a wheelchair most of the time. When he was 14 years old, cardiac hypofunction was noticed on echocardiography. When he was 17, he exhibited hypoxia and hypercapnia during sleep. At 19 years of age, β-blocker and angiotensin-converting enzyme 1 (ACE-I) treatment was initiated for cardiac hypofunction. At 21 years of age, he exhibited hypercapnia during the daytime, and he started using noninvasive positive-pressure ventilation. At 26 years of age, he was found to have thyroid cancer, and subtotal resection of the thyroid was performed. When he was 28, thyroid cancer recurred in the cervical lymph node, and lymph node dissection was performed. A physical examination when he was 31 revealed generalized hypotonia, markedly reduced muscle power in the trunk and extremities, and absence of deep tendon reflexes.

**Fig. 1:**
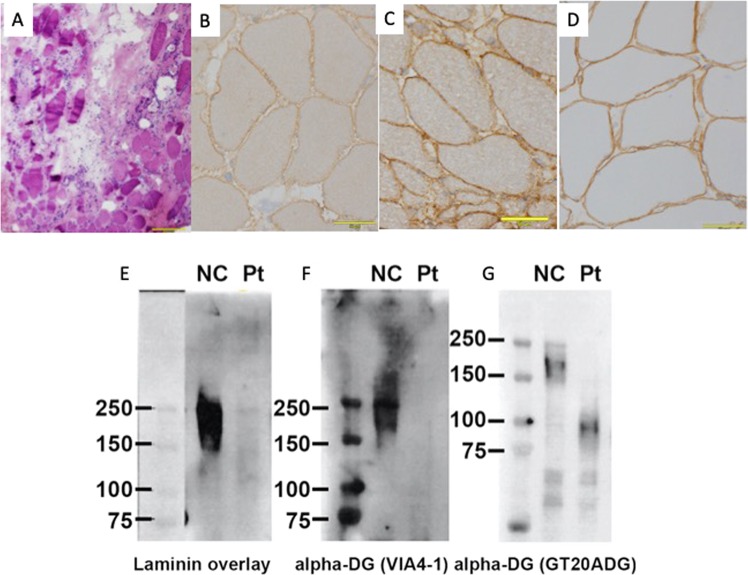
Myopathological findings, immunohistochemistry and western blot tests. **a** Haematoxylin and eosin staining of the patient’s muscle tissue at 28 years of age showed chronic myopathic changes. Extensive adipose tissue infiltration was evident, and variation in fibre size was marked. No apparent necrotic or regenerating fibres were observed. Fibres with internal nuclei were scattered. Endomysial fibrosis was marked. **b** Immunohistochemical analysis by anti-alpha-dystroglycan antibody, clone VIA4-1. The immunoreactivity to the antibody was slightly faint in the patient’s muscle surface membrane. **c** Immunohistochemical analysis by anti-alpha-dystroglycan antibody clone VIA4-1 in a control patient (no mutation in the *FKRP* gene). The immunoreactivity to the antibody was positive. **d** Immunohistochemical analysis by anti-beta-dystroglycan antibody, clone 43DAG1/8D5. The immunoreactivity to the antibody was positive in the patient’s muscle surface membrane. **e**–**g** Immunoblotting with the laminin overlay assay (**e**), anti-alpha-dystroglycan antibody clone VIA4-1 for the sugar chain of the alpha-dystroglycan (**f**), and anti-alpha-dystroglycan antibody clone GT20ADG for the core protein of alpha-dystroglycan (**g**) for normal controls (NC) and our patient (Pt). **e** The laminin overlay assay showed loss of laminin-binding activity in alpha-dystroglycan in our patient’s muscle. **f** A 156 kDa band is deficient. **g** Another 156 kDa band is deficient, and a 96-kDa band is faint. A selective loss of sugar chain immunoreactivity is suggested (**f**, **g**).

Although the clinical diagnosis was DMD, he had an undetected deletion and duplication, which was discovered using multiplex ligation-dependent probe amplification and single nucleotide variants with Sanger sequencing in the *DMD* gene at 25 years of age. A second muscle biopsy was performed when he was 28. Immunostaining of the muscle specimen revealed dystrophin positivity. Haematoxylin and eosin staining of the patient’s muscle tissue showed chronic myopathic changes (Fig. 1a). On immunohistochemistry analysis, dystrophin was present, and the result of alpha-dystroglycan was faint (Fig. 1b–d). Glycosylated alpha-dystroglycan was decreased by western blot analysis (Fig. 1e–g).

**Fig. 2:**
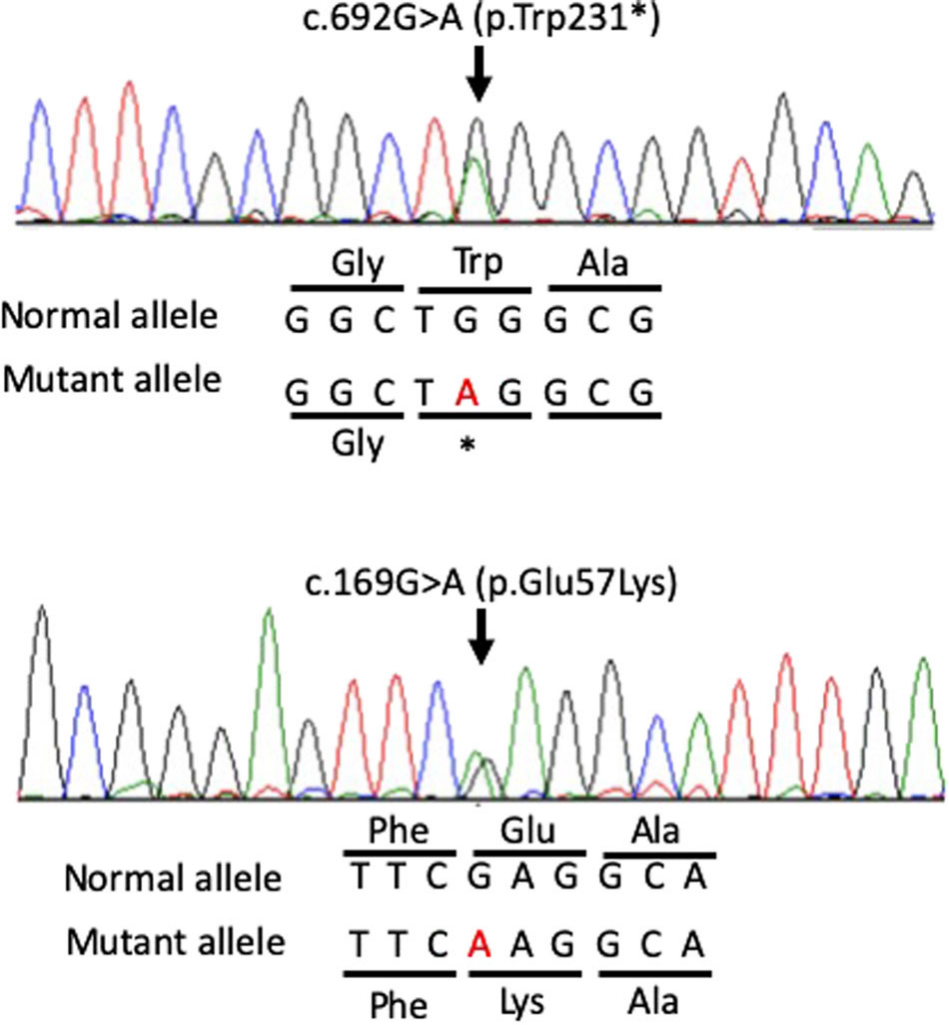
Results of FKRP gene analysis. Partial sequence chromatograms for *FKRP* in our patient.

The ethics committees of Tottori University approved the following steps of our study of this patient. The presence of pathogenic *FKRP* gene variants was confirmed by exhaustive genetic analysis with next-generation sequencing. An Illumina TruSight One sequencing panel (Illumina, San Diego, CA, USA) was performed with the MiSeq system (Illumina), followed by analysis of next-generation sequencing data, as described previously^7^. We detected compound heterozygous single nucleotide variants in *FKRP* (NM_001039885.2: c.169G>A [p.Glu57Lys] and c.692G>A [pTrp231*]) and validated their presence by Sanger sequencing (Fig. 2). These variants were inherited from the patient’s father and mother, respectively. Neither variant was described in any other databases (Exome Sequencing Project v. 6500, 1000 Genomes Project, Exome Aggregation Consortium, Human Genetic Variation, or ClinVar) as of the end of February 2019. The c.169G>A (p.Glu57Lys) variant was predicted to damage protein function, i.e., SIFT score = 0.007, Polyphen-2 score = 0.992 and Mutation Taster score = 0.9999. Additionally, his phenotype was compatible with patients with the FKRP pathogenic variant, and the following nonsense variant was detected. Applying the American College of Medical Genetics and Genomics and the Association for Molecular Pathology guidelines, we interpreted this missense variant as likely pathogenic. In another nonsense variant, c.692G>A (pTrp231*), the same amino acid change (c.693G>C [p.Trp231Cys]) had already been reported^8^.

In our patient, the manifestations of muscle weakness, hyperCKemia and cardiac and pulmonary disorders, as well as the dystrophic muscle specimens, were compatible with those observed in DMD. Patients with LGMD2I and DMD are known to have many overlapping phenotypes^9^. In other cases of limb-girdle muscular dystrophy, patients with types 2C, 2D, 2E and 2F also show DMD-like phenotypes, and these diseases are collectively known as sarcoglycanopathies. Dystrophin, alpha-dystroglycan and sarcoglycan play important roles in maintaining sarcolemma stability and muscle integrity as the dystrophin-glycoprotein complex^2^. Pathogenic variants in causative genes of sarcoglycanopathy lead to loss-of-function effects in different members of the skeletal muscle sarcoglycan complex and cause LGMD 2C, 2D, 2E and 2F^10^. However, pathogenic variants in *FKRP* lead to glycosylation defects in alpha-dystroglycan^10^. Mechanisms in each disorder are different. The correlations between the proteins may explain the similarity of the phenotype, but certain mechanisms underlying the similar clinical manifestations of DMD, LGMD2I and sarcoglycanopathies are unknown.

Our patient also suffered from thyroid cancer. Alterations in levels of glycosylated alpha-dystroglycan have been reported in leukaemia cells^11^, but the complication of thyroid cancer in patients with LGMD2I had not been reported previously, and previous reports about *FKRP* variants had not focused on thyroid cancer. The relationship between thyroid cancer in this patient and the *FKRP* variants could not be explained.

Our patient’s nonsense variant, c.692G>A (p.Trp231*), contained an amino acid change (c.693G>C, [p.Trp231Cys]), which has been previously reported^8^; patients homozygous for this missense variant have muscle–eye–brain disease. Our patient had no intellectual disability or ocular disorders, so another variant, c.169G>A (p.Glu57Lys) may produce a milder phenotype than another nonsense variant.

In conclusion, *FKRP* gene analysis should be considered for patients with muscular dystrophy, who do not have pathogenic variants of the DMD gene.

## Data Availability

The relevant data from this Data Report are hosted at the Human Genome Variation Database at 10.6084/m9.figshare.hgv.2835, 10.6084/m9.figshare.hgv.2838.
